# A novel single base pair duplication in WDR62 causes primary microcephaly

**DOI:** 10.1186/s12881-014-0107-4

**Published:** 2014-10-11

**Authors:** Verena Rupp, Sobiah Rauf, Ishrat Naveed, Windpassinger Christian, Asif Mir

**Affiliations:** Institute of Human Genetics, Medical University of Graz, Graz, Austria; Bioinformatics & Biotechnology, International Islamic University, Islamabad, Pakistan

**Keywords:** Autosomal recessive primary microcephaly (MCPH), MCPH2 locus, WDR62, Mutation

## Abstract

**Background:**

Primary microcephaly is a disorder of the brain resulting in a reduced head circumference that can come along with intellectual disability but with hardly any other neurological abnormalities.

**Case presentation:**

In this study we report on three Pakistani males from a consanguineous family with 2, 4 and 25 years, diagnosed with autosomal recessive primary microcephaly. By genotyping, Sanger sequencing and using bioinformatical approaches the disease causing mutation was identified and evaluated.

**Conclusion:**

By using a 250K SNP array, we were able to detect an 11Mb large autozygous region in the MCPH2 locus on chromosome 19q13.12. Sequencing of the associated gene, *WDR62*, revealed the frameshift causing single base pair duplication, c.2527dupG. This mutation is predicted to affect the structural features of WDR62 which in turn changes the conformation and function of the protein. Aspartic acid (D) at position 843 was found to be conserved among various ortholog species. The present findings will be helpful in genetic diagnosis of patients and future studies of WDR62.

## Background

Autosomal recessive primary microcephaly (MCPH) is a rare malformation of the head resulting in a circumference of at least 3 standard deviations below the mean for a given population, gender and age [1-3]. To date, 13 MCPH loci and their genes (MCPH1, WDR62, CDK5RAP2, CASC5, ASPM, CENPJ, STIL, CEP63, CEP135, CEP152, PHC1, CDK6 and HsSAS-6 respectively) have been mapped and identified, making this disease a genetically heterogeneous disorder, affecting 1 in 10 000 children that result from consanguineous marriages [4-17]. Recent studies in *Drosophila* revealed the influence of mutations in MCPH genes on asymmetrical cell division having an adverse effect on neuronal growth in the central nervous system during embryogenesis [18-20]. Beside a reduced cerebral cortex, a mild-to-moderate intellectual disability that strongly correlates with the head circumference, and sometimes also a delay in the linguistic development, primary microcephaly patients in general have no other developmental or neurological deficits [10,14,18,21]. Although sloping foreheads and reduced intelligence are very common, they are not listed as a basic criteria for the diagnosis of microcephaly [19,22]. The loci for MCPH2 on chromosome 19q13.12 has already been discovered in 1999 by Roberts *et al*. [23] but although excessive sequencing of this locus has been performed since then the corresponding gene, *WDR62*, remained undiscovered until only recently [5,13,16]. In human, two alternative transcripts are expressed. The full-length *WDR62* gene consists of 32 exons resulting in a genomic size of 50230bp. It encodes for a 1523 amino acid long protein that comprises 15 WD-40 repeats, one CpG signal and a polyadenylation signal [13,19,24-26]. The homodimerization region can be found on the C-terminal domain which shows no sequence homologies to any known oligomerization signals [27]. First studies on WDR62 revealed it as a JNK scaffold protein that associates with the two JNK-signalling pathway proteins JNK (c-Jun N-terminal kinase) and MKK7 (MAP kinase kinase 7) and is recruited to stress granulaes upon cellular stress induction [28]. Although Bilgüvar *et al* [5] showed in expression experiments that *WDR62* was rather a nuclear than a centrosomal protein, Bhat *et al*. [24] proved its centrosomal localization during mitosis as well as its nuclear localization but also suggested that the localization of WDR62 strongly depends on cell cycle phase and cell type. Only recently, WDR62 was identified as a phosphoprotein associated with mitotic spindle poles during prophase to metaphase transition but lacked its centrosomal localization during ana- and telophase. Depletion experiments led to a mitotic delay and to abnormal spindle formation as well as an increased formation of multipolar spindles [29].

A wide range of cortical malformations have been described for mutations in this gene, including microcephaly, pachygyria with cortical thickening, hypoplasia of the corpus callosum, polymicrogyria, simplified gyral patterns, cerebral hypoplasia, band heterotopias, lissencephlay and schizencephaly [5,13,16,17,19,24,30]. Due to the increased incidence of autozygous regions in children from consanguineous families, the probability of carrying a disease causing identical-by-decent mutation is increased in patients with autosomal recessive disorders [30]. Following this assumption, we were able to identify a novel homozygous mutation in *WDR62* in a consanguineous Pakistani MCPH2 family.

## Case presentation

### Sample collection

After obtaining informed consent, blood was drawn and DNA was isolated from 9 family members, including two affected brothers and their affected relative, according to standard protocols.

### Genotyping

Autozygosity mapping was performed with the Affymetrix GeneChip Human Mapping 250K *Nsp* Array according to the manufacturer’s protocol. The physical distance of the LOH region was determined via University of California Genome Browser UCSC [25]. Microsatellite markers in this region (D19S414, D19S220 and D19S420) were selected from ABI PRISM® Linkage Mapping Set v2.5 for fine-mapping the disease locus and to analyze the segregation among the available family members. The resulting data were analyzed with Peak Scanner Software v1.0 (Applied Biosystems).

### Sanger sequencing

*WDR62* exons and their flanking intron junctions were determined with UCSC Browser. Primers were designed using ExonPrimer (http://ihg.helmholtzmuenchen.de/ihg/ExonPrimer.html) and synthesized by Microsynth. Primer sequences are available on request.

*WDR62* exons and their flanking intron sequences were amplified with HotStarTaq Master Mix Kit (Qiagen) using a standard amplification protocol, the sequencing reaction was set up with Big Dye Terminator 3.1 (Applied Biosystems) and remaining dye nucleotides were removed with Sephadex™ G-50 superfine (GE Healthcare). Analysis of the amplicons was performed on ABI 3130*xl* Genetic Analyzer.

### Computational analysis

To predict the secondary structure of WDR62 protein, the online server Psipred [31] was used. MSA (Multiple Sequence Alignment) was performed using T-Coffee [32] to show the sequence consistency among various ortholog species of Human WDR62. Thirteen ortholog species with reference to Human (*Homo sapiens*) have been considered for this study. These species include: Chimpanzee (*Pan troglodytes*), Macaque (*Macaca mulatta*), Mouse (*Mus musculus*), Guinea Pig (*Cavia porcellus*), Megabat (*Pteropus vampyrus*), Dog (*Canis familiaris*), Opossum (*Monodelphis domestica*). Sequences of all ortholog species were collected through ensemble database.

### Findings

Five individuals of a consanguineous Pakistani family from Kotli, in the Pakistani administered Kashmir, displayed phenotypical and behavioral characteristics for primary microcephaly. An autosomal recessive inheritance was assumed according to the pedigree. DNA was available from three patients, the brothers, MCP1-2 and MCP1-5, and their more distant relative, MCP1-6.

**Figure 1:**
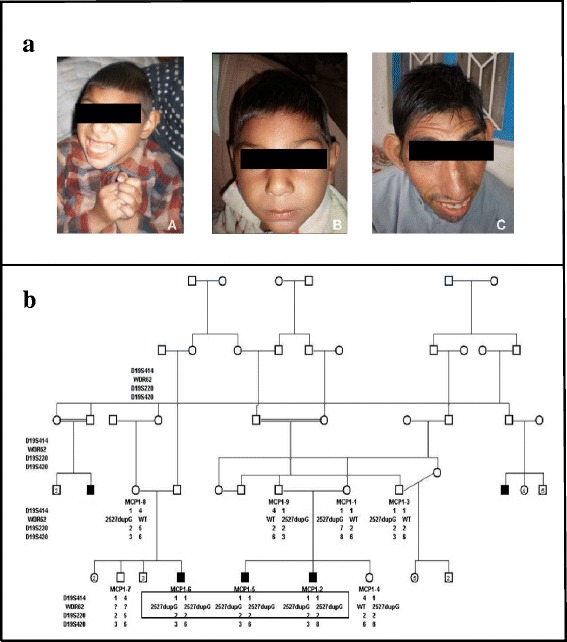
**Family Pedigree and Genotypes: a) Members of families MCP1 displaying autosomal recessive primary microcephaly:Patient MCP1-2 (A), his brother MCP1-5 (B), patient MCP1-6 (C).** **b)** Family pedigree and genotypes for 3 specific markers around chromosome 19q13.12. Affected individuals share homozygous allele 1 for D19S414 and homozygous allele 2 for marker D19S220. Relative distances in Kosambi cM were achieved from the Marshfield linkage map (http://research.marshfieldclinic.org): cen-D19S414-7.48cMWDR62-0.54cM-D1S220-4.27cM-D1S420-tel.

With 4 years and 6 months, male individual MCP1-2 (Figure 1a A) had a head circumference of only 37.47 cm and a height of 91.44 cm. He was reported to show aggressive behavior, being unable to walk due to a disabled left leg and to have an abnormally watery mouth. Beside a head circumference of only 35.94 cm and a height of 74.93 cm his 2 year old brother, patient MCP1-5 (Figure 1a B), displays no other abnormalities.

The third 25 years old patient, MCP1-6 (Figure 1a C), has a head circumference of 39.37 cm and a height of 170 cm. As for patient MCP1-2, aggressiveness and a watery mouth have been observed. The computed tomography (CT) scan of this individual revealed a reduced volume of the right cerebral hemisphere and prominent extra axial cerebrospinal (CSF) spaces with ill-defined gryal and nuclei pattern (Data not shown here). However, no local area of brain attenuation and intracerebral blood was observed. Due to the non-cooperative behavior of two other affected individuals (MCP1-2 and MCP1-5) detailed magnetic resonance imaging (MRI) scan could not be performed.

**Figure 2:**
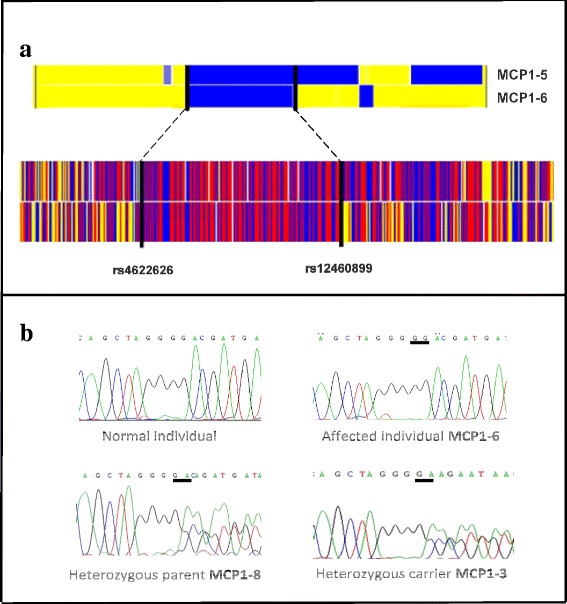
**Results of Array and Sequencing: a) SNP** ***NSP*** **250K array (Affimetrix) of Chromosome 19 of individuals (MCP1-5 and MCP1-6).** Blue and red areas indicate homozygous regions, while yellow areas indicate heterozygous regions. Patients share the same homozygous haplotype on chr. 19q12-q13.2, indicating an autozygous region. Borders are defined as the first heterozygous SNP above and under the homozygous locus. **b)** Sanger sequencing traces confirm the homozygous mutation, c.2527dupG, in all affected individuals and heterozygous mutations in the parents (underlined in black). Despite having the same microsatellite marker alleles as the affected, individual MCP1-3 is heterozygous for c.2527dupG.

Autozygosity mapping of individuals MCP1-5 and MCP1-6, revealed a single homozygous stretch on chromosome 19, reaching from q13.11 to q13.12, bounded by the SNP markers rs12460899 (29,899 Mb) and rs4622626 (40,933 Mb) (Figure 2a). Previously, the MCPH2 locus has been linked to 19q13.12, with the only recently indentified corresponding gene *WDR62* [5,13,16,23]. Fine-mapping of this locus with 3 microsatellite markers, D19S414 (31, 9 Mb), D19S220 (38,3 Mb) and D19S420 (43,7 Mb), identified a common homozygous stretch among the affected between markers D19S414 and D19S220 (Figure 1b). With *WDR62* lying at 36,545-36,596 Mb, we screened individual MCP1-6 by Sanger sequencing for mutations within this gene and revealed a homozygous duplication (insertion) in exon 22, c.2527dupG. This duplication leads to a frameshift with a premature stop codon 4 amino acids after the start of the exon (p.Asp843Glyfs*3).

**Figure 3:**
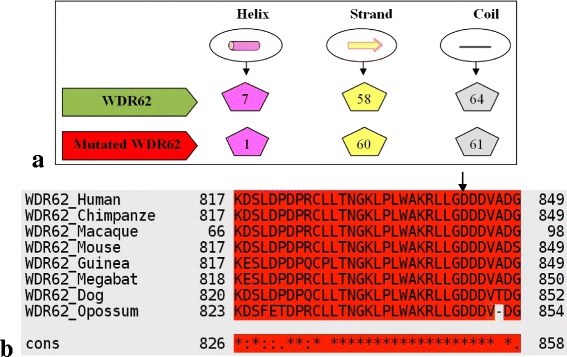
**Bioinformatics Analysis: a) Comparison of predicted numbers of structural features using Psipred for normal and mutated WDR62; b) T-Coffee Results: Multiple Sequence Alignment for Human WDR62 gene.** Conservation of amino acid D at position 843 among various ortholog species is indicated by arrow.

Segregation of the duplication in the family was confirmed by sequencing (Figure 2b). Surprisingly healthy individual MCP1-3 displays the same marker alleles as the affected but is heterozygous for the mutation in exon 22. This can be explained by the obviously non-polymorphic nature of the microsatellite markers we used for analyzing this family. Compared with the alleles of his brother, MCP1-9, a crossing over after the marker D19S414 has probably occurred. All known mutations for *WDR62*, including *the* one reported in this paper, are summarized in Table 1. According to Psipred results (Figure 3a) the mutated WDR62 structure was predicted to consist of 61 coils, 60 strands and 1 helix compared to 64 coils, 58 strands and 7 helixes in the wild type. This not only affects the conformation but also the function of the protein. T-Coffee result (Figure 3b) shows that Aspartic acid (D, replaced by Glycine as a result of the mutation) at position 843 (indicated by an arrow head) is highly conserved among various orthologous species which indicates the importance of this amino acid.

**Table 1:** **Cerebral cortical malformations causing mutations detected in** ***WDR62***

**Mutation**				
**DNA level**	**Protein level**	**Type** ^**a)**^	**Exon**	**First reported**
c.193 G > A	p.Val65Met	M	2	[13]
c.332G > C	p.Arg111Thr	M	3	[33]
c.363delT	p.Asp122Metfsx5	SD	4	[16]
c.535_536insA	p.Met179fsx21	SI	5	[24]
c.671G > C	p.Trp224Ser	M	6	[13]
c.900C > A	p.Cys300Term	N	8	[24]
c.1043 + 1G > A	p.Ser348ArgfsX63	S	8	[16]
c.1142delA	p.H381PfsX48	SD	9	[33]
c.1194G > A	p.Trp398Term	N	9	[33]
c.1198G > A	pGlu400Lys	M	9	[34]
c.1313G > A	p.Arg438His	M	10	[13]
c.1408C > T	p.Gln470Term	N	11	[5]
c.1531G > A	p.Aps511Asn	M	11	[13]
c.1576G > A	p.Glu526Lys	M	12	[5]
c.1576G > T	p.Glu526Term	N	12	[5]
c.1942C > T	p.Gln648Term	N	15	[35]
c.2083delA	p.Ser696AlafsX4	SD	17	[36]
c.2115C > G	p.Gly705Gly	CS	17	[37]
c.2527dupG	p.Asp843GlyfsX3	SI	22	Present study
c.2472_2473delAG	p.Gln918GlyfsX18	SD	23	[36]
c.2867 + 4_c2867 + 7delGGTG	p.Ser956CysfsX38	SD	23	[16]
c.2864-2867delACAG	p.D955AfsX112	SD	23	[38]
c.3232G > A	p.Ala1078Thr	M	27	[13]
c.3361delG	p.Ala1121GlnfsX5	SD	28	[33]
c.3503G > A	p.W1168Term	N	29	[33]
c.3839_3855delGCCAAGAGCCTGCCCTG	p.Gly1280AlafsX21	SD	30	[5]
c.3936dupC	p.Val1314ArgfsX18	SI	30	[13]
c.4205delTGCC	p.V1402GfsX12	SD	31	[5]
c.4241dupT	p.Leu1414LeufsX41	SI	31	[13]
c.1821dupT	p.Arg608SerfsX26	I	14	[39]

^a)^M = Missense, N = Nonsense, S = Splice-site affecting mutation, SI = small insertion, SD = Small deletion, CS = Cryptic splice site.

## Conclusion

According to Mahmood *et al*. [19] *ASPM* (MCPH5 locus) and *WDR62* (MCPH2) are the two most common genes for primary microcephaly found mutated in more than 55% of the affected families. 4% of all MCPH cases are only due to mutations in *WDR62* [35].

Due to the high prevalence of MCPH2 in primary microcephaly cases among consanguineous families more mutations in this gene will probably be revealed in the upcoming years. However, the high frequency of *WDR62* mutations in consanguineous primary microcephaly patients will especially simplify the clinical counseling and diagnostic screening of non-consanguineous primary microcephaly patients.

### Consent

We have obtained the written informed consent from the patient for publication of this case report and any accompanying images. A copy of the written consent is available for review from the Editor of this journal.
